# LMethyR-SVM: Predict Human Enhancers Using Low Methylated Regions based on Weighted Support Vector Machines

**DOI:** 10.1371/journal.pone.0163491

**Published:** 2016-09-23

**Authors:** Jingting Xu, Hong Hu, Yang Dai

**Affiliations:** Department of Bioengineering, University of Illinois at Chicago, Chicago, Illinois, United States of America; Harbin Institute of Technology, CHINA

## Abstract

**Background:**

The identification of enhancers is a challenging task. Various types of epigenetic information including histone modification have been utilized in the construction of enhancer prediction models based on a diverse panel of machine learning schemes. However, DNA methylation profiles generated from the whole genome bisulfite sequencing (WGBS) have not been fully explored for their potential in enhancer prediction despite the fact that low methylated regions (LMRs) have been implied to be distal active regulatory regions.

**Method:**

In this work, we propose a prediction framework, LMethyR-SVM, using LMRs identified from cell-type-specific WGBS DNA methylation profiles and a weighted support vector machine learning framework. In LMethyR-SVM, the set of cell-type-specific LMRs is further divided into three sets: reliable positive, like positive and likely negative, according to their resemblance to a small set of experimentally validated enhancers in the VISTA database based on an estimated non-parametric density distribution. Then, the prediction model is obtained by solving a weighted support vector machine.

**Results:**

We demonstrate the performance of LMethyR-SVM by using the WGBS DNA methylation profiles derived from the human embryonic stem cell type (H1) and the fetal lung fibroblast cell type (IMR90). The predicted enhancers are highly conserved with a reasonable validation rate based on a set of commonly used positive markers including transcription factors, p300 binding and DNase-I hypersensitive sites. In addition, we show evidence that the large fraction of the LMethyR-SVM predicted enhancers are not predicted by ChromHMM in H1 cell type and they are more enriched for the FANTOM5 enhancers.

**Conclusion:**

Our work suggests that low methylated regions detected from the WGBS data are useful as complementary resources to histone modification marks in developing models for the prediction of cell-type-specific enhancers.

## Introduction

Enhancers play an important role in temporal and cell-type-specific activation of gene expression [1]. Enhancers are short in their lengths and often consist of clusters of binding sites for transcription factors (TFs) [2]. The identification of enhancers is challenging both experimentally and computationally due to their distal locations from the transcription starting sites (TSSs) of the target genes [3]. There have been reported large-scale experimental approaches to enhancer discovery for human heart tissue [4]. The VISTA database reported 2,316 *in vivo* validated enhancers for mouse and human [5] (as of 06/09/2015). In addition, the FANTOM 5 CAGE gene expression atlas identified and characterized enhancer candidates across the majority of human cell types and tissues from the 432 primary cell, 135 tissue and 241 cell type samples [6]. However, the number of annotated enhancers (32,693 as of 04/28/2016) is far from the estimated 400,000–1 million enhancers in the human genome [7], suggesting the importance of enhancer prediction using of computational models.

The activity of enhancers has been shown to correlate with certain chromatin properties; such as accessibility of DNA to which TFs are bound and the post-translational modification in the tails of histone proteins in chromatin in the vicinity of the active enhancers [8–12]. Previous work has explored this information to build models for the prediction of enhancers [13–20]. Commonly, p300 sites overlapping with DNase-I hypersensitivity sites (DHSs), and the sites marked by H3K4me1, H3K4me2, H3K4me3 and H3K27ac, are the most explored information in the model development [13, 14, 16, 17, 21, 22]. p300, a transcriptional co-activator recruited by TF complexes, has been found at a large number of enhancers. DHSs are the accessible chromatin regions that are functionally related to the transcriptional activity. While these methods successfully identify many enhancers, the predicted enhancers tend to be bound by the known p300 binding sites. Thus, they may potentially miss out other types of enhancers. On the other hand, the analysis of whole genome bisulfite sequencing (WGBS) DNA methylation profiles has led to the identification of low methylated regions (LMRs) [23, 24]. LMRs are shown to be associated with distal regulatory elements, as they are enriched for active histone marks (e.g. H3K4me1), DHSs, p300 and TF binding sites (TFBSs) [23–26]. However, the potential of DNA methylation data for enhancer prediction has not been fully explored.

Herein, we systematically assess the usefulness of LMRs for enhancer prediction. Three questions are specifically addressed: (i) Can LMRs be used as putative enhancer sequences to build a prediction model? (ii) How is the model compared to those using information derived from p300 binding sites, DHSs, and histone modification marks? (iii) Are the predicted enhancers cell-type specific? To this end, we propose a novel framework, namely, LMethyR-SVM, which builds a model for enhancer prediction from sequences of cell-type-specific LMRs based on a weighted support vector machine. Although LMRs are enriched for marks of active regulation, not all sequences of LMRs are necessarily enhancers. For this reason, LMRs are initially called unlabeled. To dissect the LMRs, *in vivo* validated enhancer sequences from the VISTA enhancer database [5] are explored to divide the unlabeled set into a reliable positive set, a likely positive set and a likely negative set, representing our confidence in the LMRs being putative enhancers. By encoding sequences based on a *k*-mer scheme, this division is achieved according to the density ranks of LMRs on a density distribution estimated from the LMRs that overlap with the VISTA enhancers. With a random negative set, the weighted support vector machine (wSVM) is then designed to learn a prediction model by best dividing the unlabeled set of the LMRs into three datasets and assigning the associated weights. LMethyR-SVM trains on sequences of cell-type-specific LMRs and predicts enhancers by scanning the genome without using information of DNA methylation. We compare the performance of LMethyR-SVM models built on the LMRs from the H1 human embryonic stem cell type (H1) and the fetal lung fibroblast cell type (IMR90) with existing methods. We further investigate the cell-specificity of the predicted enhancers.

## Materials and Methods

**Fig 1:**
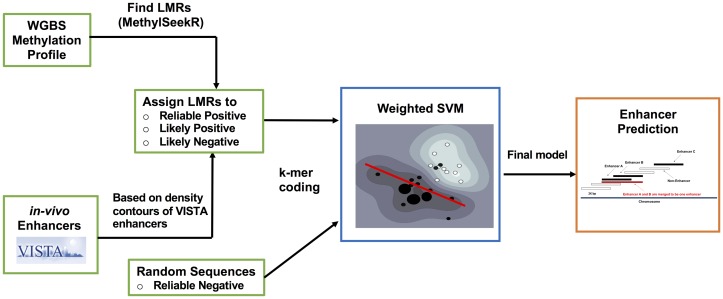
The proposed framework of LMethyR-SVM.

Our framework, LMethyR-SVM, begins with a DNA methylation profile obtained from a specific cell type. Then the cell-type-specific LMRs were identified and the LMRs that overlap with the VISTA enhances were used to estimate the density distribution of enhancers in the *k*-mer space. Subsequently, each LMR sequence was assigned a rank based on the distribution. Finally, the wSVM learns a model from the LMRs and a random background set by determining the best division of the unlabeled LMR set into a reliable positive (RP) set, a likely positive (LP) set, and likely negative (LN) set. The proposed framework of LMethyR-SVM is shown in Fig 1.

### Low methylated regions (LMRs) in H1 and IMR90 cell types

The WGBS data of DNA methylation in H1 and IMR90 cell types are available from the website of the Salk Institute for Biological Studies [27]. The WGBS data were generated using the MethylC-seq technique and the raw data have been mapped and quantified for the methylation level at individual cytosines in the genome. The downloaded data are processed methylcytosine profiles. We lifted the data to the UCSC hg19 assembly using liftOver provided at the UCSC Genome Browser [28]. LMRs were identified using the Bioconductor package MethylSeekR [24] with the recommended parameters. MethylSeekR identifies hypomethylated regions as stretches of consecutive CpGs with methylation levels below a fixed threshold and further divides them into unmethylated and low methylated regions. Genome regions with SNPs, and X and Y chromosomes were excluded from the analysis since the LMRs could not be reliably detected. More details about this procedure can be found in the previous publications [24, 25].

### The VISTA enhancers

The VISTA Enhancer Browser is a central resource for experimentally validated human and mouse noncoding fragments with gene enhancer activity in transgenic mice [5]. The 967 validated human enhancers were downloaded from the VISTA database (as of 6/09/2015). The VISTA enhancers are highly conserved in other vertebrates or rich for epigenomic evidence (ChIP-Seq) of putative enhancer marks. Although these enhancers are not specific to a cell type, the subsets that overlap with the LMRs from H1 cell type and IMR90 cell type were identified respectively as putative cell-type-specific enhancers.

### Weighted support vector machine (wSVM)

LMRs are cell-type-specific, although there may be some overlaps between the LMR sets generated from two different cell types. As suggested in the previous studies, LMRs are indicative of distal active regulatory regions that may include enhancers [23–26]. However, the functional role of LMRs in gene regulation is unclear. Since they may also include some sequences that are not relevant to enhancers, the set of LMRs is initially called unlabeled (U), instead of being labeled as a positive set of enhancers in our study. From this unlabeled set, the LMRs were further divided into three sets: RP, LP, and LN according to their ranks of density on the estimated density distribution (the procedure described in the next section). The rank indicates the degree of resemblance to the LMRs that overlap with the VISTA enhancers. To facilitate machine learning, a reliable negative (RN) set of sequences was constructed by randomly selecting loci that do not include the sequences of the UCSC exons and the LMRs. The shuffle function in BEDTools was used to randomly permute the genomic locations of a feature BED file (i.e., LMRs) on the human genome (hg19). The tool excludes loci of exons and LMRs while generating a set of sequences that preserves the distribution of length and number on each individual chromosome as those of the LMRs. In addition, the random sequences containing base ‘N’ besides ‘ATGC’ were excluded. All sequences were represented using a *k*-mer encoding scheme, i.e., each sequence is encoded by a vector *x* with 4^*k*^ entries, each of which indicates the number of times that a particular *k*-mer appears in the sequence. In this work, we used *k* = 5 based on our preliminary investigation in wSVM training. Furthermore, each vector *x* was normalized by dividing the sum of all entries in *x*.

To reflect our confidence in the given sets of RP, LP, and LN of LMRs, the wSVM model was proposed as the following optimization problem.

$$\begin{array}{l} minimize_{w,\xi ,b}\frac{1}{2}{∥w∥}^{2}+C_{+}^{RP}\sum_{i\in RP}\xi_{i}+C_{+}^{LP}\sum_{i\in LP}\xi_{i}+C_{-}^{LN}\sum_{i\in LN}\xi_{i}+C_{-}^{RN}\sum_{i\in RN}\xi_{i}\\ \begin{array}{ll} subject\;to\;y_{i}\left(w^{T}x_{i}+b\right)\geq & 1-\xi_{i}\;\left(i=1,2,\ldots ,n\right)\\ & \xi_{i}\geq 0\;\left(i=1,2,\ldots ,n\right) \end{array} \end{array}$$ (1)

where *ξ*_*i*_ is a slack variable that allows for error for misclassification, and *n* is the total number of training examples. For each *x*_*i*_, it is labeled as *y*_*i*_ = 1 if it is in RP or LP; and *y*_*i*_ = −1 otherwise. The weights $C_{+}^{RP},\;C_{+}^{LP},C_{-}^{LN}\;and\;C_{-}^{RN}$ are weight parameters for wSVM to penalize misclassified training examples in RP, LP, LN, and RN, respectively. However, the best division of LMRs together with the weights have to be learned using a cross-validation procedure described below. We restrict $C_{+}^{RP}\geq C_{+}^{LP}$ since we have more confidence in the reliable positive set RP than in the likely positive set LP. Consequently, a sequence from RP receives a larger penalty than a sequence from LP if it is classified as negative. Similarly, we set $C_{-}^{RN}\geq C_{-}^{LN}$ for the reason that we have more confidence in RN than in LN and misclassification of a sequence from RN as positive receives a larger penalty than if a sequence from LN is misclassified as positive class.

### Partition of an unlabeled set of LMRs based their ranks on a density distribution

The following scheme was used to determine our confidence for each sequence in the unlabeled set of the LMRs. The LMRs overlapping the VISTA enhancers were considered as the putative enhancers in that cell type. This subset of LMRs was used to generate an estimated underlying density distribution of the sequences in the *k*-mer space. The overlapping VISTA enhancer sequences themselves were not used because they contain primers of several hundred base pairs on the flanking regions, which may bring “noise” into model training. The R package pdfCluster [29] was used to detect the underlying cluster structure of the sequences based on the kernel density estimation. In contrast to traditional clustering methods based on metric of distance or dissimilarity, the density-based clustering method associates cluster(s) with the regions around the modes of the density underlying the data [30]. By measuring the density of an LMR to its assigned density center in the *k*-mer space, the sequence was given a rank. The higher its rank is, the closer a sequence is to its cluster center. We normalized the ranks and used the corresponding quantile values in the following procedures. LMRs that were ranked at the top *δ* quantiles were labeled as RP; those under 15% quantile were labeled as LN; and the remainder were labeled as LP. The best value of *δ* was learned through the cross-validation (CV) procedure in wSVM training (more detail in Supporting Information S1 File).

### Protocol for training and testing

The wSVM model described above can be solved as an optimization problem if a quantile threshold *δ* for dividing the LMRs into RP and LP, a quantile threshold to determine LN, and a set of weights $C_{+}^{RP},\;C_{+}^{LP},C_{-}^{LN}\;and\;C_{-}^{RN}$ are given. To determine the best values for these parameters, a 5-fold CV procedure was used to choose a group of models with the highest cross-validated *F*-scores. The parameter set that generated the model with the highest precision from the group was chosen as the best parameter set. Here *F*-score is defined as

$$F\;=\;\frac{2*precision*recall}{precision+recall}$$ (2)

$$where\;\;recall=\frac{TP}{TP+FN}\;,\;\;precision=\frac{TP}{PP}$$ (3)

and *TP* and *FN* are the numbers of LMRs in RP and LP that are predicted as positive and negative, respectively; and *PP* is the number of LMRs that are predicted as positive. Note that the definition of *F*-score seems to be identical to the one used in standard binary learning, where a golden positive set is available. However, it is different here in the sense that it uses RP and LP sets. Therefore, the best model cannot be chosen merely based on *F*-score, rather, determined by the one with the highest precision among the models with the best F-scores. In addition, genomic coverage, as an additional constraint in model selection was added. Genomic coverage is defined as the fraction of nucleotides that are covered by the predicted enhancers in the hg19 human genome. In the training process, the genomic coverage of the predicted enhancers on Chromosome 1 was used as a proxy for the entire genome. In the CV process, models that predict enhancers with genomic coverage more than a prescribed threshold value were excluded. With this additional constraint, we could effectively avoid generating models that predict a large fraction of the genome as enhancers. The details of parameter tuning and the cross-validation procedure can be found in the Supporting Information S1 File, S1 and S2 Figs. Libsvm [31] was used to train wSVMs with linear kernels, and the heuristic procedure was implemented in shell scripts.

The enhancer prediction was carried out by sliding a window of size 2 kb for every 500 bp on the human genome and a score is assigned to each window by the final wSVM classifier. Windows with positive scores were predicted as enhancer windows. Overlapped enhancer windows were joined and the covered region was reported as one enhancer (We later called these enhancers ‘concatenated enhancer’); the covered region may be reported for more than one enhancer by separating at any possible negatively scored windows in it, a situation can occur by the way of window sliding. Note that the choice of 2 kb window size for prediction was the result based on the comparison with other window sizes, i.e., 500 bp (for every 100 bp), 1 kb (for every 200 bp) and 1.5 kb (for every 500 bp). We found that even with the use of smaller window sizes for prediction, the median lengths of the concatenated enhancers are greater than the one obtained from the 2 kb window size. This result is consistent with the previous publications that a sliding window of size 2 kb is the most appropriate choice [7, 13, 17].

### Validation procedure for the predicted enhancers

The publically available data on epigenetic marks associated with enhancer activities were used in the validation procedure. Our criteria are similar to the ones used in RFECS [13] and DELTA [18] but more stringent. Specifically, a true positive marker (TPM) set was defined as any of the DHS, CBP/p300 and enhancer-associated TF sites if the site is located 1 kb away from the nearest TSS. We used the highest SVM score enhancer window to represent the concatenated enhancer where it resides for the validation procedure, since the use of the concatenated enhancers inflates the results. An enhancer was labeled as one of the three categories: “validated”, “misclassified” or “unknown” by the following criteria.

1. If the nearest TPM lies within 1 kb of a predicted enhancer, then the enhancer is “validated”.
2. If the nearest TSS lies within 2.5 kb of a predicted enhancer, and the nearest TPM is greater than 1 kb away, then the enhancer is “misclassified”.
3. If there is neither a TPM within 1 kb nor a TSS within 2.5 kb of the enhancer, then the enhancer is “unknown”.

The “validated” enhancers were divided into 6 mutually exclusive states as in [13]: “p300+/-DHS”, “DHS only”, “TF+DHS”, “TF only”, “TF+p300”, “p300+DHS+TF”. For example, “p300+DHS+TF” means that an enhancer is validated by all the three markers; “p300+/-DHS” includes “p300+DHS” and “p300”. The percentages of the predicted enhancers that were labeled as “validated”, “misclassified” and “unknown” were calculated.

The experimental data of DHSs and p300 binding sites for the two cell types, and enhancer-associated TFs, i.e., NANOG, CEBPB, TEAD4 for H1 and CEBPB for IMR90, were downloaded from “ChIP-Seq Experiment Matrix” from the ENCODE website [32]. The p300 binding sites for IMR90 were obtained from RFECS [13]. The “uniform”, or “narrow peak” type of the ChIP-Seq data for each marker was selected. These types of data were consolidated from multiple experiment results and were controlled at a significantly small false positive rate. Meanwhile, since the total length/coverage of the sites are small, the validation by overlapping these markers is unlikely due to randomness.

### The FANTOM 5 enhancers

The 32,693 enhancers reported from FANTOM5 –the Functional Annotation of the Mammalian Genome project were downloaded from the consortium website (as of 04/28/2016) [33]. The *in vivo* active enhancers are identified based on the distinct bidirectional CAGE (Cap Analysis of GENE Expression) pattern using the FANTOM5 panel of tissue and primary cell samples [6]. The FANTOM5 CAGE expression atlas encompasses 432 primary cell, 135 tissue, and 241 cell type samples from human and annotated 32,693 enhancers across the majority of human cell types and tissues. Although neither H1 nor IMR90 cell type is included, the FANTOM enhancers are used as a proxy of active enhancers to check the extent of enrichment in the predicted enhancers. A predicted enhancer window is considered to be overlapping with a FANTOM enhancer if the overlap is at least by 1 bp.

### Statistical tests

All statistical test results for proportional difference and enrichment were obtained using z-test in R.

## Results

**Fig 2:**
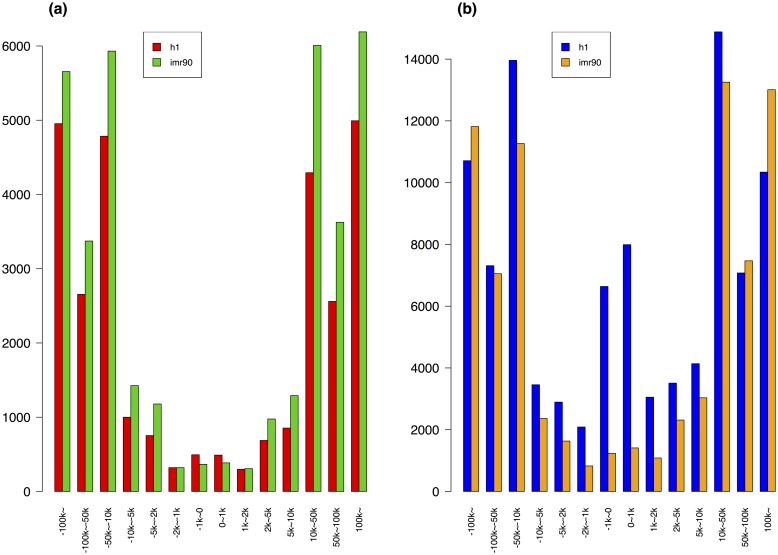
The distributions of the LMRs and predicted enhancer windows. (a): The distributions of the LMRs to the nearest TSSs; red for H1 and green for IMR90. (b): The distribution of all the enhancer windows to their nearest TSSs; blue for H1 and brown for IMR90. The distance was measured from the center point of a sequence to its nearest TSS.

As described previously, MethylSeekR was used to identify LMRs from H1 and IMR90 WGBS methylation profiles. It found 29,138 and 37,033 LMR sequences for H1 and IMR90, respectively. The median length of LMRs is 410 bp in H1 and 622 bp in IMR90. As expected, they mostly reside in distal regions that are more than 2 kb away from their nearest TSSs (Fig 2(a)). In addition, only 9.2% and 44.2% of LMRs overlapped with the p300 binding sites in H1 and IMR90, respectively. The higher percentage of the overlap in IMR90 may be due to a much larger number of p300 binding sites (52,878) in IMR90 in comparison to that in H1 (8,934). The LMR sequences with lengths less than 200 bp or greater than 3 kb were removed for the subsequent model training, resulting in 23,017 and 34,174 LMR sequences in H1 and IRM90, respectively.

We found that 106 LMRs overlap with 105 VISTA enhancers for H1 and 129 LMRs overlap 117 VISTA enhancers for IMR90 by at least 1 bp. These LMRs are called the VISTA-overlapping LMRs. Next, we applied pdfCluter to derive an estimated nonparametric density distribution based on the VISTA-overlapping LMRs in each cell type. This distribution was used to assign each LMR a rank according to its density. The ranks were normalized to quantile values. High quantile values correspond to high densities. The LMRs with quantile values below 15% for H1 and 11% for IMR90 were assigned to the LN set respectively based on our preliminary analysis. Then, the 5-fold CV procedure was applied to find the best quantile cutoff to divide the rest of the LMRs into sets of RP and LP, and the corresponding weights in wSVM for each cell type. The details can be found in Supporting Information S1 File. We chose genomic coverage at 5% in our model to report analysis results, although models at 10% and 15% of genomic coverage were also developed. The value of 5% was determined because the number of the predicted enhancers with this constraint generated a similar number of enhancers as other methods so that a fair comparison could be made. The quantile cutoffs between RP and LP learned from wSVM were both 95% for H1 and IMR90.

A summary of the predictions is given in Table 1. The distributions of the genomic locations of all the enhancer windows with respect to the nearest TSSs are shown in Fig 2(b)). It can be observed that the majority of them are distal to the nearest TSSs. Interestingly, the distribution remained almost the same for the enhancers predicted from the models that were trained after removing LMRs overlapped with promoter regions, defined as 2 kb up- and down- stream of the TSSs (S3 Fig in Supporting Information S1 File).

**Table 1:** The summary of the predicted enhancers obtained from LMethyR-SVM.

	Number of enhancer windows	Number of enhancers^(a)^	Estimated genomic coverage^(b)^	Median length of enhancers	Maximum length of enhancers
H1	98,045	34,437	3.67%	2.5 kb	66 kb
IMR90	77,762	35,203	3.05%	2.5 kb	113 kb

^**(a)**^ Enhancers are the continuous regions obtained by merging all overlapping enhancer windows and separated into two at non-enhancer windows if exist.

^**(b)**^ The genome coverage is the estimate from Chromosome 1.

The numbers of the predicted enhancer windows are 98,045 and 77,762 in H1 and IMR90, respectively. We obtained the predicted enhancers by merging the overlapping enhancer windows. This resulted in 34,437 and 35,203 enhancers in H1 and IMR90, respectively. The genome coordinates of the predicted enhancers for H1 cell type and for IMR90 cell type are provided in Supporting Information S2 and S3 Files, respectively. Since some of the predicted enhancers are quite long, e.g., the longest enhancer in IMR90 is of 113 kb, we further selected the highest-scored enhancer window from each enhancer as the representative of this enhancer for a fair comparison with other methods.

### The performance of LMethyR-SVM

**Fig 3:**
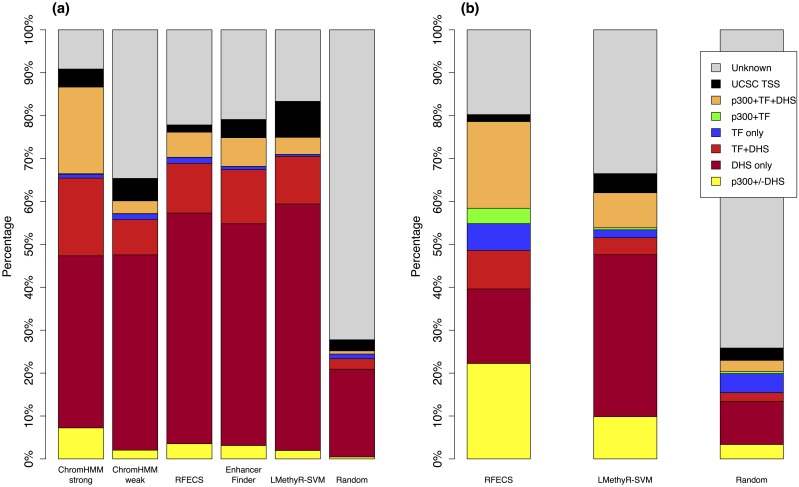
Results of comparison with other enhancer prediction models. (a) for H1 and (b) for IMR90. “Validation” rates were computed as percentages of overlaps with either DHSs, p300 sites or enhancer-associated transcription factor binding sites (NANOG, CEBPB and TEAD4 for H1 and CEBPB for IMR90); “Misclassification” rates were computed as percentages of overlaps with the UCSC annotated TSSs. “Validated” enhancers can be further divided into one of the mutually exclusive categories: “p300+/-DHS”, “DHS only”, “TF+DHS”, “TF only”, “TF+P300”, “p300+DHS+TF”. For LMethyR-SVM, the highest-scored enhancer windows were used. The total numbers of the enhancers in H1 predicted from the individual methods are 17,828 (ChromHMM Strong), 217,350 (ChromHMM weak), 54,121 (RFECS), 37,263 (EnhancerFinder), 34,437 (LMethyR-SVM) and 34,437 (Random). The total numbers of the enhancers in IMR90 predicted from the individual methods are 82,392 (RFECS), 35,203 (LMethyR-SVM) and 35,203 (Random).

Since there is no true set of cell-type-specific enhancers for validation, we evaluated the predicted enhancers from three aspects. We first compared our results with those obtained from three state-of-art enhancer prediction methods: ChromHMM [20], RFECS [13], and EnhancerFinder [16]. We also used a random set of sequences to serve as a baseline for comparison, which was generated by randomly selecting 2 kb windows to be enhancers with the same number as our enhancers. We also compare the predicted enhancers with FANTOM enhancers. The three methods utilize various types of chromatin modification information coupled with supervised or unsupervised machine learning models. However, none of them has used epigenetic modification information of DNA methylation. Since it would be ideal for fair comparison if the lengths of predicted enhancers were similar, we chose the highest-scored enhancer windows from our prediction in this validation analysis. The results of the predicted enhancers at genomic coverage 5% based on the validation procedure using the TPMs are shown in Fig 3. Note that for IMR90, RFECS is the only tool that has made prediction besides LMethyR-SVM. In general, a high percentage of “validated” and low percentages of “misclassified” and “unknown” suggest high confidence in the prediction results. These criteria allow the performance comparison among different prediction models with relatively small bias since a common set of experimental markers is used.

ChromHMM [20] used a hidden Markov model to segment the genome of 200 bp windows into multiple states on the basis of consensus on patterns. Then, domain experts manually annotated each state. ChromHMM integrated most of the existing ChIP-Seq data on chromatin modifications. It predicted enhancers for each of the 9 different cell types: H1, GM12878, Hepg2, Hmec, Hsmm, Huvec, K562, Nhek, and Nhlf. These data are available from the Annotation Database on the UCSC Genome Browser website. For H1 cell type, ChromHMM predicted 235,178 enhancers, which are further cataloged into 217,350 “weak enhancers” and 17,828 “strong enhancers”. Since ChromHMM used the ChIP-Seq data from ENCODE for model learning, it is not surprising that it is one of the best performers, especially for its “strong enhancer” category (Fig 3(a)). Its validation rate (86.65%) is significantly higher than that of LMethyR-SVM (74.87%, *P* < e-15). However, its “weak enhancers” category has far too many enhancers, resulting in a worse validation rate (60.14%, *P* < e-15) compared to LMethyR-SVM (74.87%).

The second method, RFECS, was trained with a random forest learning model on 24 ChIP-Seq histone modification data from ENCODE and the p300 binding data for IMR90 were generated from their own study. RFECS makes the prediction using sliding windows of 1.5 kb to 2.5 kb. It has predicted 54,121 enhancers for H1 and 82,392 for IMR 90 [13]. However, the published enhancers are in the form of a 1bp locus. Therefore, we expanded this locus by 1 kb on both sides for fair comparison. The validation rate of the RFECS prediction is 76.14% for H1, indicating that LMethyR-SVM has a slightly worse performance (74.94%, *P* = 2.6e-5) (Fig 3(a)). Similarly, RFECS for IMR90 has a validation rate of 78.60%, which is better than LMethyR-SVM (62.02%, *P* < e-15) (Fig 3(b)). The performance of RFECS could be due to its extensive training based on the comprehensive ChIP-Seq data and due to the use of the p300 data generated by their own study in the validation procedure.

The third method, “EnhancerFinder”, uses a multiple kernel learning approach to integrate DNA sequence motifs, evolutionary patterns, and 2,469 functional genomics datasets generated by the ENCODE project and other small scale studies [16]. The tool predicted enhancers for multiple cell types (including H1) and multiple organs, but not for IMR90. The published 82,490 H1 enhancers have a validation rate of 74.87%, which is not significantly different compared with LMethyR-SVM (74.94%, *P* = 0.41) (Fig 3(a)).

In summary, the performance of LMethyR-SVM evaluated using TPMs is highly encouraging, considering the facts that only DNA methylation data were used in building the prediction model and all the other methods relied on the abundant ChIP-Seq experiment data of histone modifications, and sites of p300 binding and DHSs. The way of training and evaluation by all the other models could favor towards the validation of their results and may lead to overestimated performance. On the other hand, the experiment data of the DNA methylation used in LMethyR-SVM is independent of any of the ENCODE experiment results. This suggests that LMRs is informative as a source to build enhancer prediction models.

Next, we set out to examine the extent of overlaps between the predicted enhancers made by LMethyR-SVM and ChromHMM for H1; by LMethyR-SVM and RFECS for IMR90 in order to evaluate the novel putative enhancers predicted by LMethyR-SVM. ChromHMM has predicted the largest number of enhancers since it used the comprehensive ENCODE chromatin datasets based on an unsupervised method [20]. In addition, we also analyzed the overlap with the FANTOM5 enhancers. We used the predicted enhancer windows from our method in this analysis. The results are summarized in Table 2. This part of the analysis was performed only on the predicted enhancers that were validated with the TPMs. From Table 2 we observe that 11.79% of the enhancer windows predicted by LMethyR-SVM in H1 overlap with the FANTOM5 enhancers, which is significantly higher than that of the ChromHMM enhancers (4.67%, *P* < e-15). Similarly, 14.24% of the enhancer windows predicted by LMethyR-SVM in IMR90 overlap with the FANTOM5 enhancers, representing a significantly higher proportion than that of RFECS (13.78%, *P* = 0.013). This result for IMR90 is interesting because RFECS has a better performance than LMethyR-SVM in terms of the validation rate based on the TPMs in IMR90. Further, we took the enhancers that are uniquely predicted by each method and compared the enrichment for the FANTOM enhancers. It can be seen that 10.01% of the enhancer windows uniquely predicted from LMethyR-SVM in H1 overlap with the FANTOM5 enhancers, representing a significantly higher proportion than that of ChromHMM (4.12%, *P* < e-15). However, the enhancer windows uniquely predicted from LMethyR-SVM in IMR90 have a lower proportion (8.63%) for the FANTOM5 enhancers compared to that of RFECS (12.35%, *P* < e-15). Finally, the overlapped enhancers generated from the two corresponding methods in each cell type always included a significantly higher number of the FANTOM5 enhancers compared to the enhancers unique to each individual method (data not shown). This analysis provides further evidence indicating that LMRs may provide a complementary sequence profile other than the regions marked by histone modification, p300 binding and DHS sites for enhancer prediction.

**Table 2:** The summary of the overlap between the predicted enhancers and the FANTOM5 enhancers.

		Unique ^(a)^(%FANTOM^(c)^)	Total ^(b)^(%FANTOM)
H1	ChromHMM	120,539(4.12%)	146,172(4.67%)
	LMethyR-SVM	45,629(10.01%)	75,947(11.79%)
IMR90	RFECS	55,665(12.35%)	64,765(13.78%)
	LMethyR-SVM	32,775(8.63%)	51,146(14.24%)

^**(a)**^ Unique: the number of the enhancers that were uniquely predicted by a method and validated by TPMs.

^**(b)**^ Total: the total number of the enhancers that were predicted by a method and validated by TPMs.

^**(c)**^ %FANTOM: the percentage of the enhancers that overlap with the FANTOM5 enhancers.

Finally, we investigated if the LMethyR-SVM predicted cell-type-specific enhancers. This task is challenging because no complete set of validated enhancers exist for each cell type. Therefore, we evaluated the cell-type-specificity indirectly by comparing the LMethyR-SVM predicted enhancer windows for H1 with those predicted by ChromHMM for H1 and 8 other cell types (H1, GM12878, Hepg2, Hmec, Hsmm, Huvec, K562, Nhek and Nhlf). Our argument is that if the overlap between the LMethyR-SVM and the ChromHMM enhancers in H1 is higher than that in any of the other 8 cell types, it may suggest cell-type-specific enhancers were predicted by LMethyR-SVM. Indeed, it is observed that the LMethyR-SVM predicted enhancer windows have the highest percentage of overlaps (39.2%) with the ChromHMM enhancers for H1 cell type (Table 3). The proportion of overlap in H1 cell type is significantly higher than that in any of the other 8 cell types (26.5%–37.6%, P<e-15).

**Table 3:** The comparison of the proportions of overlap between the enhancer windows predicted by MethyR-SVM in H1 and ChromHMM annotated enhancers in 9 cell types.

Cell type	# enhancers predicted by ChromHMM	# overlaps between LMethyR-SVM and ChromHMM enhancers ^(a)^	Proportion of overlap
H1	235,178	38,397	0.392
GM12878	236,344	25,934	0.265
Hepg2	201,190	29,365	0.300
Hmec	283,718	36,805	0.376
Hsmm	267,423	30,743	0.314
Huvec	228,423	30,347	0.310
K562	242,306	32,598	0.332
Nhek	272,728	28,908	0.295
Nhlf	235,293	27,493	0.280

^**(a)**^ The number of enhancer windows predicted by LMethyR-SVM in H1 overlapped with those predicted by ChromHMM.

### The evolutionary conservation in the predicted enhancers by LMethyR-SVM

Enhancers that are active during the early mammalian developmental stage tend to be conserved [34]. H1 cell type is one of the embryonic stem cells derived from the inner cell mass of a blastocyst, an early-stage pre-implantation embryo. In addition, most of the VISTA enhancers are ultra-conserved non-coding DNA sequences in the vertebrate genomes when first being selected for testing. Indeed, 99.8% of the VISTA enhancers overlap with at least 1 phastCons46ways conserved segments at vertebrate level. PhastCons is a computational tool that finds and annotates conserved segments on the human genome using comparative genomic information. The conservation information was downloaded from the track on the UCSC website [28]. We wanted to check if our prediction model, which uses information on the VISTA enhancers, reflects this inclination of conservation. Since there are about 5 million conserved segments annotated by PhastCons on the entire human genome, they are densely populated, a window of 2 kb in length can easily include one conserved element by chance. To avoid over-estimation, each predicted enhancer was represented by its midpoint and any enhancer overlapping with exons has been removed for this analysis. If the midpoint locates on any conserved segment, then the enhancer is considered “conserved”. We used the predicted enhancer windows from our method in this analysis. A random set was also included to serve as the baseline for comparison.

**Fig 4:**
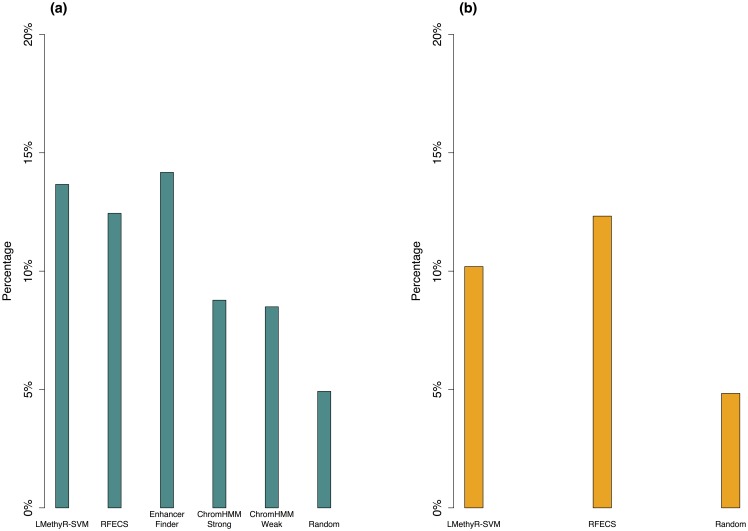
Comparison of the conservation levels for the predicted enhancers. Proportions of overlaps between the predicted enhancers from each method with the conserved segments by the UCSC *PhastCons46Ways* conservation annotation at vertebrate level. Each enhancer is represented by its midpoint (1bp); (a) for H1 and (b) for IMR90.

It is shown that for H1 cell type the predicted enhancers obtained from LMethyR-SVM have the second highest proportion of enhancers that overlap with at least one conserved segment (Fig 4(a)), only after EnhanerFinder, which also has integrated the VISTA enhancer information. ChromHMM may predict the most heterogeneous type of enhancers due to its non-supervised model built on a comprehensive dataset. It shows moderate but above the conservation level of the random set. There is no significant difference in conservation between its “strong enhancers” and “weak enhancers” (data not shown). For IMR90 cell type, the RFECS predicted enhancers are slightly more conserved than those of LMethyR-SVM (Fig 4(b)). Together, LMethyR-SVM has predicted the enhancers that tend to be more conserved in H1 cell type, which may be more relevant to the early developmental stage in H1 cell type.

Taken all together, LMethyR-SVM is effective in learning from cell-type-specific LMRs for the prediction of enhancers. The predicted enhancers are largely distinctive compared to those predicted from other models in H1. The indirect evidence suggests that the predicted enhancers may be cell-type-specific. In conclusion, the LMRs derived from a WGBS DNA methylation profile may be used as complementary information to predict enhancers missed by other models.

## Discussions

We designed a novel framework (LMethyR-SVM) for the prediction of enhancers using cell-type-specific low-methylated regions (LMRs) detected from the whole genome bisulfite sequencing (WGBS) data. Our rationale is based on the observation in previous work that LMRs are potential distal active regulatory regions that may include enhancers [24, 25, 35]. The unique feature of our approach is that the training set is built on the single source of epigenetic profiles of DNA methylation, contrasting to other methods that rely on data of multiple epigenetic profiles, such as large scale ChIP-Seq studies of histone modification, co-activator p300 binding and DNase—I hypersensitive sites. LMethyR-SVM only uses LMRs for model training and it does not depend on DNA methylation profile for prediction, while some of the other methods also require ChIP-Seq data for prediction. We did not combine other histone modification data in our model because we wanted to evaluate the potential of the DNA methylation profiles, more precisely LMRs, in the prediction of enhancers. Using methylation data from the H1 human embryonic stem cell type (H1) and the fetal lung fibroblast cell type (IMR90), we have compared ours performance with several state-of-art predictive models (ChromHMM [20], RFECS [13], and EnhancerFinder [16]). The validation using “true positive marker” (TPM) shows that our model has a considerably good performance in terms of validation and misclassification rates among some of the best methods. We further found that a large proportion of the TPM validated enhancers were not predicted by ChromHMM in H1 and were more enriched for the FANTOM enhancers, indicating LMethyR may have captured enhancers that were missed by ChromHMM. Although we were unable to directly validate cell-type-specificity of the predicted enhancers by LMethyR-SVM in H1, we provided indirect evidence through comparing with the enhancers predicted by ChromHMM for other 8 cell types. In addition, we found that only 48% of our predicted enhancers are overlapped by at least 1 bp from the two cell types of H1 and IMR90. Our results collectively support that WGBS data may be used as a complementary type of data for identification of cell-type-specific and tissue-specific enhancers.

Few work has explored methylation profile for the development of enhancer prediction model, except the one that uses the methylation profile generated from the Illumina Infinium HumanMethylation450 BeadChip [36]. The array-based profile represents only a very small percentage of CpG sites in the genome. In that work, the sequence features of neighboring regions of the selected methylated CpGs that are negatively correlated with the expression level of predicted target genes were used to develop a prediction model. By contrast, WGBS methylation data can provide comprehensive information on the methylation including low methylation regions. It should be noted that the bisulfite treatment also converts 5-hydroxymethylated cytosines. Therefore, the WGBS methylation data cannot distinguish between 5-methylated cytosines and 5-hydroxymethylated cytosines [37]. Therefore, it is not clear which mechanism of methylation regulates the activity of the enhancers predicted from LMRs.

Enhancers have very different GC content in the genomic background [38], which could be used to construct a negative set to improve model performance. Our inspection on the LMR sets of H1 and IMR90 shows the median GC content (proportion of nucleotide ‘G’+proportion of nucleotide ‘C’) of the sequences to be 47.6% and 46% and the corresponding negative sequences’ to be 41.1% and 41%, respectively. The predicted enhancer sequences have even a higher GC content of 56.2% for H1 and 52.5% for IMR90, although we did not use information of GC content specifically. For further investigation, we built a negative set with similar GC content as that of the positive set and trained the model in the same fashion. We found that the validation rates were reduced by 15%–20% for both cell types, meanwhile, the misclassification rates were also reduced by several percentages. This result seems to indicate that the use of a negative set adjusted by GC content could increase the precision of prediction at the cost of reduced sensitivity. Overall, our analysis shows that enhancers have different GC content compared to the genomic background and this signal may be implicitly encoded in the k-mers. We also note while some previous publications built a negative set with similar GC content [19, 39], others did not [15, 16, 18, 40].

The enhancers predicted from our method are generated by merging overlapping enhancer windows. The median length of the enhancers ranges between 2.5 kb to 3 kb, and the maximum length can be as long as ~113 kb, suggesting that they may be super-enhancers. Super-enhancers are large clusters of transcriptional enhancers playing essential roles in gene expression regulation [41]. Recently, several databases of super-enhancers such as SEA [42] and dbSUPER [43] were released based on the analysis of large scale ChIP-Seq for TFs and H3K27ac modification data in modENCODE [44], ENCODE and Human Epigenome Roadmap [45]. We checked the overlap between our predicted enhancers with the super-enhancers in dbSUPER for H1 and IMR90 (not available in SEA). The minimum, median and maximum lengths of super-enhancers are 876 bp, 9,590 bp and 62,270 bp for H1 and 4,664 bp, 25,650 bp, 110,800 bp for IMR90, respectively. We found that 43% (295 out of 684) and 68.9% (346 out of 502) of the super-enhancers overlap with at least one LMethyR-SVM predicted enhancer windows in H1 and IMR90 respectively, indicating the considerable proportions of overlap. A comprehensive evaluation will be further required to delineate the enhancers predicted from LMRs. It will be possible in near future as WGBS profiles will accumulate along with RNA-Seq data, histone modification marks, DNase-I hypersensitive sites, p300 binding sites and other cell-type-specific TF binding sites in multiple cell types and tissue/organs. In addition, it would be interesting to investigate if better prediction models can be obtained by combining information on ChIP-seq histone modifications and DNA methylation profiles in future.

Various machine learning algorithms have been proposed to derive enhancer prediction models using epigenetic profiles other than DNA methylation. They include support vector machines [15, 16], artificial neural network (CSI-ANN) [17], random forest (RFECS), a combination of Gibbs sampling and linear regression [19], hidden Markov model (ChromHMM), a two-step tissue-specific SVM model (EnhancerFinder), ensemble approach (DELTA) based on AdaBoosts [18], and a recent method using diverse data sources from the ENCODE histone marks data, VISTA and the FANTOM enhancers (DEEP) [40]. The most successful methods built their models based on extensive ChIP-Seq experiment data for the model training. DEEP has been shown to out-perform others due to its usage of multiple types of data from ENCODE, VISTA and FANTOM. We did not choose to compare with DEEP, since our primary goal is to reveal whether the LMRs from WGBS DNA methylation profiles have the potential for enhancer prediction.

The designed learning framework was motivated by our early work of unlabeled-positive learning for text mining [46] and unlabeled-negative learning for prediction of MHC class II peptide binding [47], and a recent work for disease gene prediction in which an unlabeled-positive learning framework was designed based on a weighted support vector machine [48]. The commonality in the three applications is the existence of a true positive set in the unlabeled set. By contrast, in the current work, there is no golden truth available for enhancers for a specific cell type. Our choice of the nonparametric procedure (pdfCluster) was effective in retrieving a reliable set of LMR sequences (i.e., reliable positive set). This was carried out based on the ranks on the density distribution estimated on the LMRs that overlap with the VISTA enhancers using pdfCluster. LMethyR-SVM also benefited from the design of the wSVM learning model by best separating the unlabeled LMR set into reliable positive, likely positive and likely negative sets.

## Supporting Information

S1 Fig (TIF)

S2 Fig (TIF)

S3 Fig (TIF)

S1 File Supporting materials of procedures and results. (PDF)

S2 File The predicted enhancers by LMethyR-SVM for H1 cell type. (XLSX)

S3 File The predicted enhancers by LMethyR-SVM for IMR90 cell type. (XLSX)
