# Correction: LMethyR-SVM: Predict Human Enhancers Using Low Methylated Regions based on Weighted Support Vector Machines

**DOI:** 10.1371/journal.pone.0165551

**Published:** 2016-10-20

**Authors:** Jingting Xu, Hong Hu, Yang Dai

The following information is missing from the funding statement: This work was partially supported by the UIC Research Open Access Article Publishing (ROAAP) Fund. The funder had no role in study design, data collection and analysis, decision to publish, and preparation of the manuscript.

## References

[1] Xu J, Hu H, Dai Y (2016) LMethyR-SVM: Predict Human Enhancers Using Low Methylated Regions based on Weighted Support Vector Machines. PLoS ONE 11(9): e0163491 doi: 10.1371/journal.pone.0163491 27662487 10.1371/journal.pone.0163491 PMC5035071

