# Supplementary figures and images for: LMethyR-SVM: Predict Human Enhancers Using Low Methylated Regions based on Weighted Support Vector Machines
Source: PLoS One. 2016 Sep 23;11(9):e0163491. doi: 10.1371/journal.pone.0163491 (PMC5035071; doi:10.1371/journal.pone.0163491)

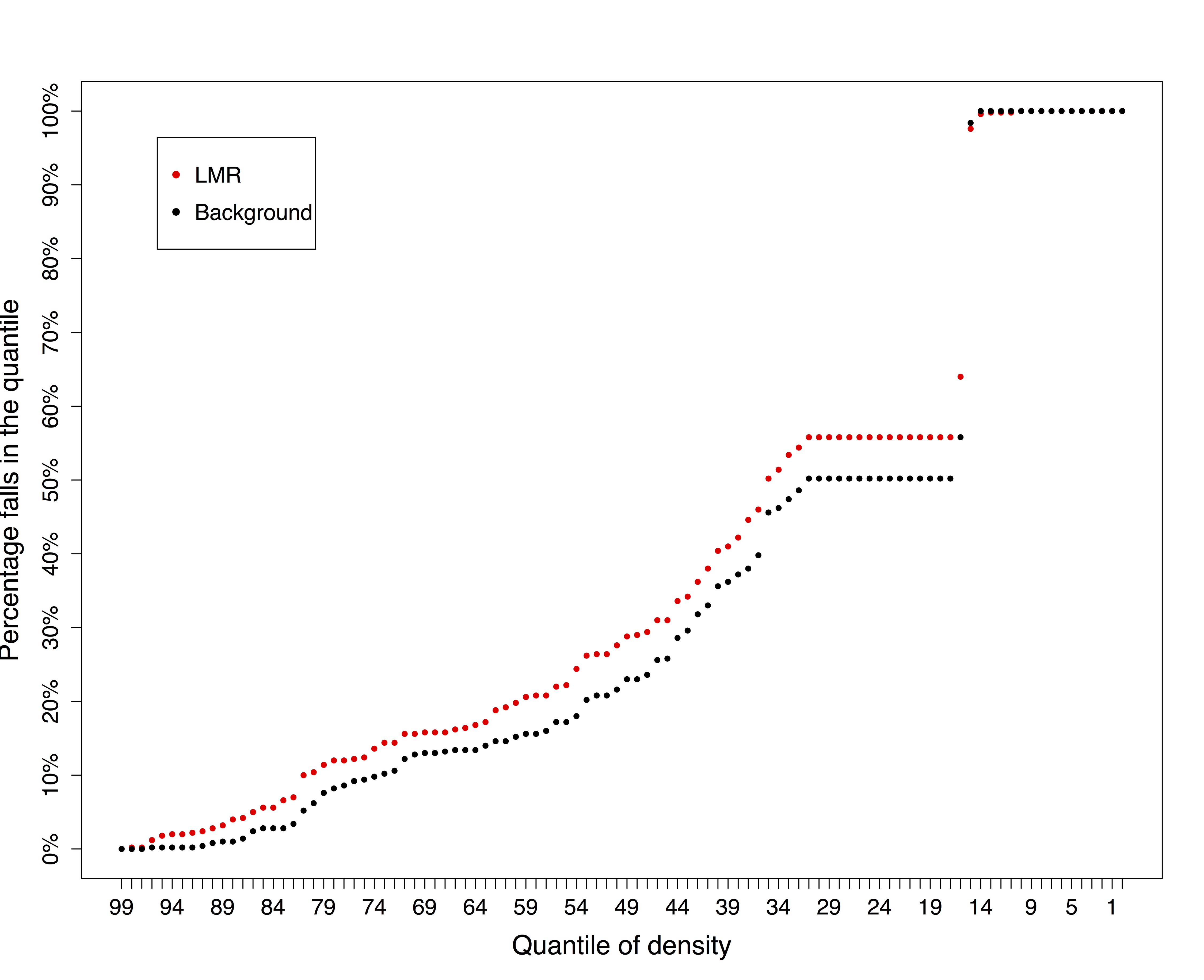

Supplement: S1 Fig — (TIF) [file pone.0163491.s001.tif]

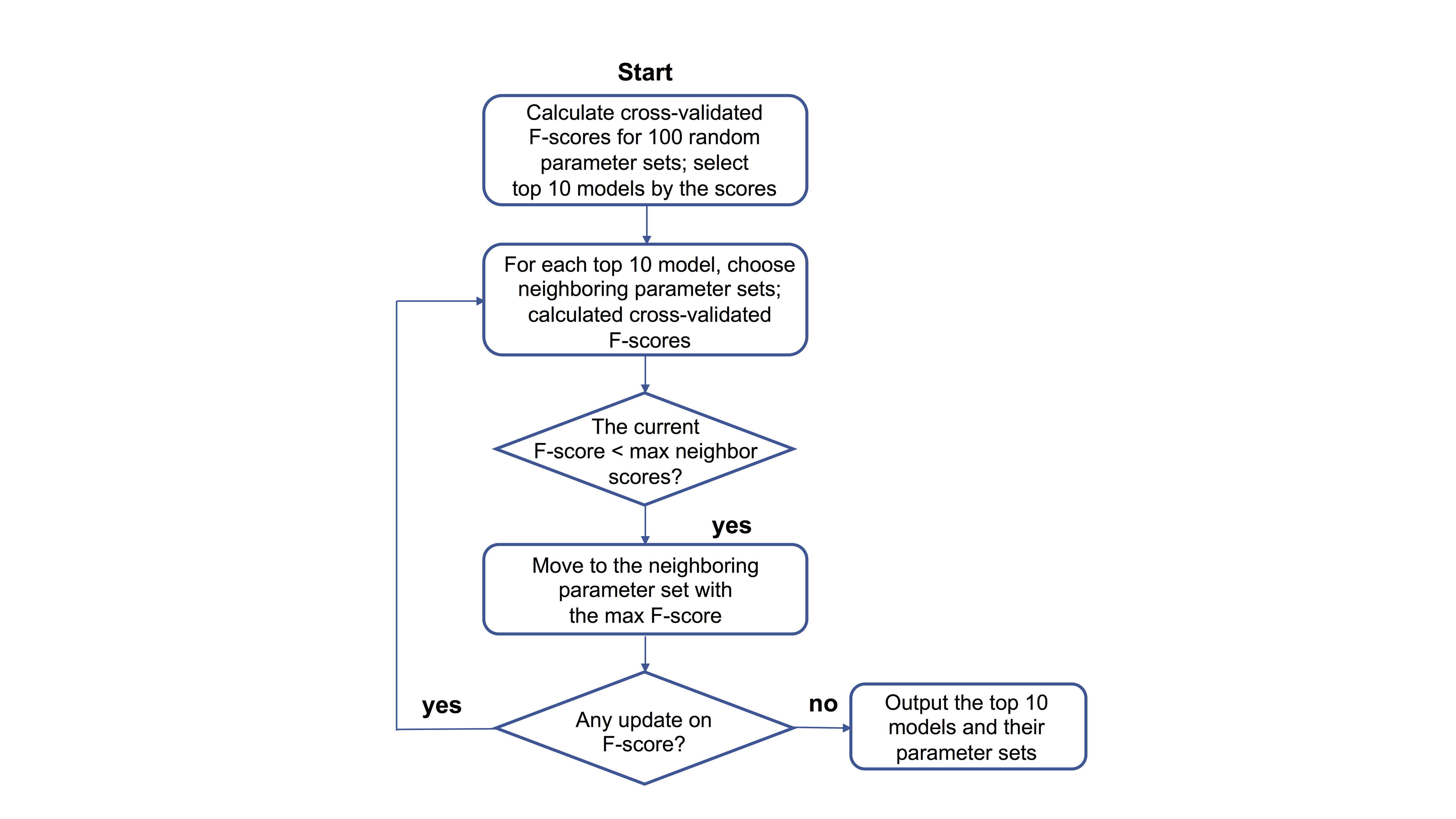

Supplement: S2 Fig — (TIF) [file pone.0163491.s002.tif]

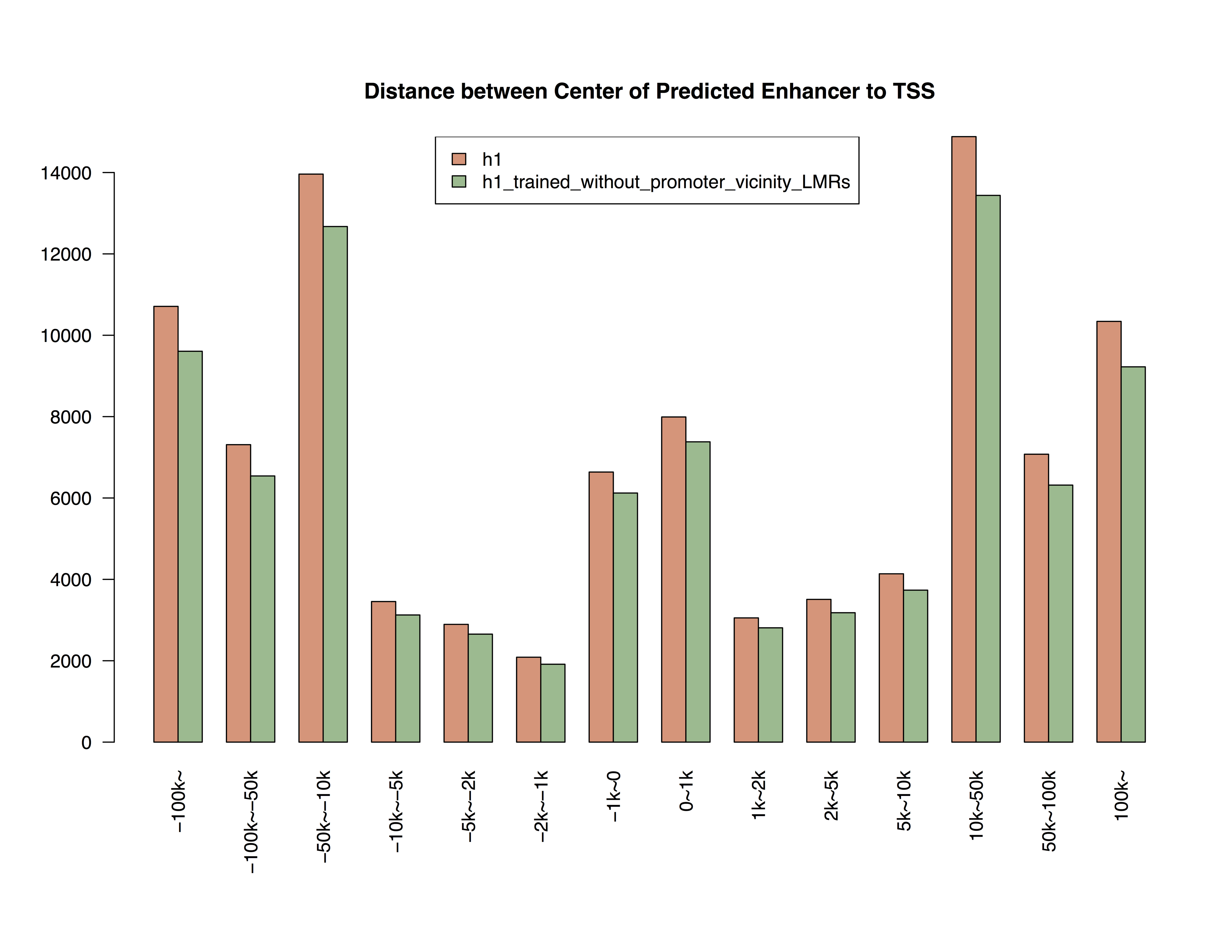

Supplement: S3 Fig — (TIF) [file pone.0163491.s003.tif]
