# Supplementary material for: LMethyR-SVM: Predict Human Enhancers Using Low Methylated Regions based on Weighted Support Vector Machines
Source: PLoS One. 2016 Sep 23;11(9):e0163491. doi: 10.1371/journal.pone.0163491 (PMC5035071; doi:10.1371/journal.pone.0163491)
Supplement: S1 File — (PDF) [file pone.0163491.s004.pdf]

LMethyR-SVM: Predict human enhancers using low methylated regions based on weighted support vector machines

Jingting Xu[1*], Hong Hu[1*], Yang Dai[1*§]

[1] Department of Bioengineering, University of Illinois at Chicago, Chicago, Illinois, United States of America

Supporting Information (S1 File)

The procedure for dividing LMRs into RP, LP, and LN sets

The R package “pdfCluster” was used to perform the clustering analysis on the LMRs that overlap with the VISTA enhancers to estimate the underlying clustering density distribution of enhancers in the feature space of *k*-mer. pdfCluster performs cluster analysis based on a nonparametric estimation of the density of the observed variables [1]. Functions are provided to encompass the whole process of clustering, including kernel density estimation, clustering and subsequent graphical diagnostics. Due to the limitation of pdfCluster, we could only test with $k=3$. Therefore, each LMR sequence was represented as a point in the $4^3=64$ dimensional feature space. With the estimated density distribution, each point (i.e., LMR) was assigned a rank based on the distribution and was subsequently normalized the ranks to a quantile value. High quantile values indicate that the points densely clustered with other points and low quantile values indicate that the points sporadically scattered in the feature space. PdfCluster also identifies sets of points at same density in the feature space. The projection of the density distribution onto any two dimensional space shows contour curves similar to those in topographic maps.

We assume that sequences with high quantile values contain more informative motifs/features for their high occurrences of common motifs. On the contrary, sequences with low quantile values are those away from all high density spots so they do not carry as much common motif/feature information. To verify this assumption, we assigned ranks to the background sequences based on the same density distribution for H1 cell type. We compared the sets of the LMRs and the background for their quantile values.

Since both the LMRs and background sets are large, we bootstrapped sequences to make an approximation. We randomly selected from 500 to 5,000 sequences from both sets, excluding the 106 LMRs that were used to estimate the density distribution and examined the quantile values of the sampled sequences. Results are shown in **S1 Fig**. We observed that at a high quantile value (x-axis), the proportion of LMRs (y-axis) at the quantile value or greater is higher than that of the background, which means in general, LMRs are more likely to occur in high density clusters than background sequences in the feature space. We also observed at quantile ~15% and less, LMRs and background sequences reach the same proportion.

Based on this information, LMRs with quantile values below 15% were assigned to LN (likely negative) as at this quantile, LMRs and background sequences were not distinguishable from their density status. The rest of LMRs were assigned to the category of RP (reliable positive) if they have quantile values above a threshold value δ, otherwise, the category of LP (likely positive). The threshold δ was determined by the cross-validation procedure for the values of 55%, and 65% - 95% with a 5% increment. For IMR90 the quantile cutoff value below 11% was applied to assign LMRs to LN based on the similar analysis.

The heuristic procedure for parameter searching in wSVMs

Since the ratio between the values can reflect the magnitude change and has approximately the same model performance from our inspection, we fixed the value of C_{+}^{RP} to be 2^{9}, and let C_{+}^{LP} vary from 2^{-9} to 2^{8}, C_{-}^{RN} from 2^{-9} to 2^{9}, and C_{-}^{LN} from 2^{-9} to the largest value without violating the constraint $C_{-}^{RN} > C_{-}^{LN}$. With the varying values of C_{+}^{LP}, C_{-}^{RN} and C_{-}^{LN}, we searched the 3D grid space with a grid point in the space representing a set of parameter values and used F-score as the cross-validation (CV) criterion with a given genomic coverage as a constraint. The genomic coverage was estimated from the predicted enhancers on chromosome 1 by the model for convenience. If the coverage exceeds the constraint, then the weight set is not considered.

The heuristic search strategy works as follows. For a quantile threshold δ, we randomly selected 100 grid points, i.e., combinations of weight values, and computed the average *F*-score value from the 5-fold CV for each of these 100 points. After the first round, we selected the 10 grid points with which models generated highest F-scores. Then we carried out second round of search from these 10 points in their neighbors. We computed *F*-scores at all neighbors of each point in the grid space. If the highest F-score from a neighbor is greater than that of the current point, then we replaced the current point by this neighbor, i.e., moved to the point. Otherwise, no move was made from the current point. This search was continued until none of the current top 10 points could be replaced anymore. The final top 10 points (i.e., sets of weight values) are the output of the heuristic procedure (**S2 Fig**). The best weight values for the given quantile threshold was chosen from the model that has the highest precision among the top 10 models. Then, we repeated this procedure for all quantile cutoff values in the range specified above. The best parameter set is the one that generates the highest precision at the given genomic coverage. The corresponding quantile cutoff value was used to divide the LMRs into RP and LP sets for the final model.

Weight parameters in the final model

We further looked at the weights assigned to LN in the final models selected by the cross-validation procedure in H1. They are extremely small compared to those assigned to RP, LP, RN, indicating that the contribution of the LMRs whose quantile ranks below 15% to the final prediction model is negligibly small. Thus, the wSVM model designed in our

framework has successfully identified those LMRs with low similarity to the highly confident putative enhancers and prevented them from participating in the final model.

References

1. Menardi G, Azzalini A. An advancement in clustering via nonparametric density estimation. Statistics and Computing. 2014;24(5):753-67.

S1. Fig. Density vs. Proportions. The x-axis shows the quantile and y-axis shows the proportion of LMR or background sequences below a quantile. At different quantile cutoff (x-axis), higher proportion of LMRs falls in the quantile than that of the background sequences.

S2 Fig. The heuristic procedure for searching the best quantile cutoff values and the weight values.

S3 Fig. The distributions of the distance of the predicted enhancers to their nearest TSSs in H1 cell line. The brown color represents the enhancers predicted from the original LMRs; The green color represents the enhancers predicted from the LMRs after removing those LMRs whose center points overlap with the promoter regions (2 kb up- and down-stream of TSSs).
